# Observations on the Pathology of Fever, with Strictures on the Physiological Doctrine of Broussais

**Published:** 1830

**Authors:** John Eberle

**Affiliations:** Professor of Materia Medica & Obstetrics in the Jefferson Medical College


WESTERN JOURNAL

OF THE

MEDICAL & PHYSICAL SCIENCES.

APRIL, MAY, & JUNE, 1830.

### ESSAYS AND CASES.

Art. 1.—*Observations on the Pathology of Fever, with Strictures on the Physiological Doctrine of Broussais.*—*By* John Eberle, M. D. *Professor of Materia Medica & Obsterics in the Jefferson Medical College.*

The history of practical medicine consists of little else than a review of the doctrines which have successively risen and sunk again, concerning the nature and treatment of fever. Whatever other objects of interest or importance within the dominion of medical science may have attracted the attention of physicians, fever has at all times been viewed as presenting the most extensive and inviting field for observation and the exercise of ingenuity. It is in this department that observation and research have been most industrious in accumulating materials, and that hypothesis has luxuriated in her wildest exuberance.

When indeed it is considered that the destroying angel has made his most desolating visitations under the form of febrile epidemics; and that in the long list of human maladies *fever*

occurs in perhaps nine cases out of ten, the paramount importance of this subject is strongly forced upon our convictions.*

From a restrospective glance at the history of our science, we are forced to acknowledge that there is perhaps no subject which is more eminently calculated to humble the pride of human reason than this one. For, in relation to this subject especially, pathology has been in a continued state of revolution and instability. The human mind has been engaged with this subject for near three thousand years. Theories have risen and sunk again in a continued and rapid series of succession; each has had its hour "to strut upon the stage," and its votaries to yield it faith; but the stream of time has hitherto overturned all these insubstantial, though often highly wrought fabrics.

Has the mind then made no real advancement in relation to the pathology of fever? Are we now no nearer correct and rational views concerning this important subject, than were our forefathers? Has genius always wandered in idle quest, and brought back no substantial trophies from the regions of pathological speculation on this point? Far from it. Like the asymptotes of the parabola, the human mind is continually verging towards the point of truth, although it may never reach it in relation to the essential nature of fever. There has probably never been any theory or doctrine promulgated on this subject which did not clear away some old rubbish, or bring to fuller view some of the relations of the phenomena it presumed to elucidate. The dreams of speculation have vanished; but the facts and correct principles which

*"If we except," says Van Swieten, "those who perish by a violent death, and such as are extinguished by mere old age, and which are indeed few, almost all the rest die either of fever, or of diseases accompanied with fever. We read in Pliny with what fear and trembling the Romans endeavored to have this universal disease—*fever*, appeased by their supplications in the temple of Fanum. And hence perhaps it is that fevers are called *diseases* by Hesiod, and that Horace calls all diseases simply fevers when they rushed out of the box of Pandora—

'Post ignem ætherea domo
Subductum, Macies, et nova febrium
Terris incubuit cohors.'

*Van Swieten's Com.* vol. v. p. 1.

were necessarily mingled with them, remain as so much valuable treasure saved out of the wrecks of former systems. The mass of solid materials which has been thus gradually accumulated, has now in a great measure displaced those vague and hypothetical foundations upon which former doctrines in relation to this subject were constructed. Hypothesis is no longer tolerated in science. Philosophy does not acknowledge her as a legitimate servant. The cyclus of her empire has gone by; and the genius of rational induction is now the only power under whose direction the votary of science presses forward to conquest in the fields of knowledge.

Like many other things which are at once obvious to the senses, and concerning the existence of which almost every one can decide, *fever* does not admit of a strictly correct and unobjectionable definition; since there is not a single symptom which is invariably present, and which can be regarded as absolutely essential to its existence.

Boerhaave collected together, from a great number of authors, all the symptoms which had been observed in fevers. He then struck from this list, all those symptoms which do not appear in all, but only in certain particular modifications of fever—retaining such only as by the common consent of authors and his own observations, were found to be present in every instance of fever. The result was, that only three symptoms were left standing—namely, a quick and frequent pulse—preternatural heat of the surface of the body—and a sense of cold or chilliness in the commencement. But he might have gone further, and struck from his list these symptoms also; for it is quite certain that cases of fever do occur in which there is neither preternatural quickness and frequency of the pulse, nor an increased temperature of the surface of the body; nor is a sense of chilliness, though perhaps the most constant of all the febrile symptoms universally present in the initial stage of fever.

Notwithstanding the great difficulty, or rather impossibility of giving a strictly unexceptionable scientific definition of *fever*, yet the train of phenomena which this state of disease presents under all its modifications—varying more or less in their concomitance and succession—offers, upon the whole, a character sufficiently distinct and definite for easy and certain recognition.

Pathologists have divided fevers—according to the mode of their development—into *idiopathic* and *symptomatic*, and the propriety or impropriety of this division constitutes, at the present day, one of the most important, and warmly contested subjects in pathology. By the former class are understood those fevers which are developed and sustained by causes, which produce a general morbid state of the system, independent of a local inflammation or fixed irritation in some part of the system. Those who admit the existence of such fevers, suppose that the remote febrific cause produces a deleterious impression on the sentient extremities of the part upon which it acts, which deranging function after function, according to the catenation of the organic sympathies, will finally result in a state of general disease, characterized by the ordinary phenomena of fever; or, as they presume, the remote cause may gradually change the healthy character of the circulating blood, which, acting as a morbific irritant on the heart and arteries, will give rise to febrile reaction.

Many highly eminent pathologists, on the contrary, contend that such fevers can have no existence; and that all febrile excitement is purely symptomatic, and of course essentially and wholly dependent on a pre-established local irritation or inflammation. According to these views, the direct influence of the remote causes of fever is limited to the production of the primary local inflammation or irritation, the subsequent pyrexial phenomena being the result solely of this primary local affection; in other words, the secondary and sympathetic excitement of the pre-established focus of irritation. At the head of those who advocate the exclusive symptomatic nature of fever is Broussais, who, whatever may be thought of his peculiar doctrines in relation to this subject, has manifested a professional zeal, an activity and acuteness of intellect, which have justly placed him high among the "greater lights" of our profession.

Not satisfied however with the adoption and defence of the general doctrine of the universality of *symptomatic* fever, Broussais contends that the inflammation or irritation whence the febrile sympathies radiate as from a focus, is almost universally located in the mucous membrane of the alimentary canal; and hence *gastro-enteritis* is with him the *fons et origo* of febrile phenomena.

That fever is a very common result of local inflammation, is unquestionable. So intimately are all the various parts of the animal body connected with each other by the ties of sympathy, that no structure or organ can be strongly irritated without causing a sympathetic irritation in other organs or structures. If the primary irritation involve the sanguiferous capillaries, the irritation will be communicated by sympathy to the general vascular system, and fever will be the result; but if the local irritation be purely nervous, it is diffused, and as it were locked up in the general nervous system, and the result will be convulsions, or some other form of general nervous affection. Without doubt too, inflammation of the mucous membrane of the alimentary canal is much more common in febrile diseases than was formerly, and by many is still supposed. It is even probable that in many instances of fever, such an inflammation exists as the *primary* and essential cause of the febrile phenomena. This may be especially the case in those instances of fever which result from the combined agencies of impure and indigestible diet and atmospheric vicissitudes. But although we may admit the correctness of these observations, yet to refer all fevers, remitting, intermitting, and continued, to a *gastro-enteritis*, as is done by Broussais and his followers, is as remote from truth as it is detrimental in its influence on practice.

The advocates of the *physiological* doctrine, as it is called, endeavor to support their sentiments in relation to this subject, both by the phenomena which are detected on post-mortem examination, and by arguments founded on physiological principles. It is affirmed that marks of inflammation occur, almost universally, in the mucous membrane of the alimentary canal, in subjects that die of febrile affections. The capillary vessels, to a greater or less extent, of this membrane, it is said, are found injected; and in many instances other and less equivocal traces of previous inflammation are found on dissection. Admitting that such manifestations of inflammation are as universal as they are asserted to be, is there not reason to conclude, that very frequently at least, the inflammation thus detected, *supervened* during the course of the disease, as a *consequence* of the fever, rather than that the inflammation was pre-established, and became the immediate exciting cause of the febrile phenomena? We frequently see inflammations supervene in parts exposed to observation many days after general fever has been fully established. Indeed, when it is considered that in all febrile affections, the secretions which are poured into the intestinal tube are unnatural and vitiated—that the process of digestion is suspended, and consequently that fermentation and decomposition of the contents of the stomach and bowels are especially favoured—is there any cause to wonder that we should so often meet with traces of inflammation in the digestive organs in those who die of febrile affections? The Broussaian mode of treating fevers, although especially meant to obviate such inflammations, appears to me in one respect, well calculated to favour their occurrence. The almost total proscription of purgatives from the list of our remediate means for the treatment of fever, so far from lessening the tendency to gastro-enteritis, tends, I conceive, in general, to an opposite result. In a recent work by Bouillaud, there are upwards of sixty cases of fever reported, in not a single instance of which was there a purgative medicine administered by the mouth. In all of these cases however marks of inflammation, and in the majority ulcerations were detected in some portion of the mucous membrane of the bowels. That this should have been observed, will not appear strange, when it is considered that in all these cases, most of which continued from three to four weeks, all the acrid and vitiated contents of the intestines were suffered to remain, undisturbed, to act on their delicate lining membrane.

Nothing but the blind zeal which usually characterizes the disciples of a *new doctrine* could, one should suppose, induce any one to withhold a laxative under the apprehension of its causing injurious irritation, and yet suffer, without any such fears, the most irritating substances to lie quietly in the bowels. It is true, laxative *lavements* were repeatedly resorted to in these cases, but that these did not disturb or remove the acrid materials which were enclosed in the bowels, is abundantly manifest from what Mr Bouillaud himself has stated. After having gravely told us that in all the cases he describes, the traces of mucous inflammation in the bowels were *tres prononce*, he states that, "in general the stomach and small intestines were *filled with a yellowish or greenish bile, and that the residue of the alimentary substances which were found in the small and large intestines, invariably exhaled an intolerably fetid smell, and frequently exhibited the consistence of mustard. This residue, mixed with various fluids secreted in the intestines, appeared to have undergone a complete process of putrefactive decomposition, as was evident from the extreme offensiveness of the smell, and the fetid gas which distended the bowels.*"*

Can it be reasonably supposed that the transient and moderate irritation of a purgative in these cases would have been more injurious than the constant impressions of the acrid and irritating substances which were so long left in immediate contact with the bowels? It is thus, it can hardly be doubted, that many instances of gastro-enteritis, so abundant in the practice and dissections of the Broussaian school, are developed. Were laxatives employed with due moderation, it is probable, that the so much dreaded *gastro-enterite* would in some instances at least, perhaps in many, be prevented, and the world deprived af a large proportion of those triumphant demonstrations which are continually brought out in formidable array against the unbelievers of the *physiological doctrine*.

As a further offset to the evidence adduced from post mortem examination, it must be observed, that so far as the mere red-

*Traite Clinique et Eperimental des Fievres. Par J. Bouillaud, Paris, 1826.

ness or injected state of the mucous membrane is concerned, we can draw no certain inference as to the previous existence of inflammation in this structure. That these phenomena are frequently the result of changes effected in *articulo mortis*, or *post mortem*, is fully demonstrated by the observations of Mr. Yellowly and of Mr. Seeds.†

---

†"It must have happened to every one," says the former of these writers "accustomed to the examination of dead bodies, to see appearances of vascular injection in the villous coat of the stomach. Such appearances have very frequently been referred to inflammation, but they have probably been but little studied. I have several times been present at the examination of bodies, where the vascularity of the villous coat of the stomach was so considerable as even to give rise to suspicions that the appearance had been produced by something deleterious. I was therefore induced to embrace frequent opportunities of viewing the state of the inner surface of the stomach, and I so often found in it the appearances alluded to, as to induce me to imagine, that the opinion which is commonly entertained of their being marks of disease, is not well founded. In persons suddenly destroyed, when apparently in perfect health, he found the mucous membrane of the stomach highly injected." Mr. Seeds too found that in animals bled to death, the membraneous structures frequently exhibit a state of injection which might, at first sight, be readily mistaken for inflammation.

It is well known that the arterial tubes possess a power of contracting to a considerable extent, by what Bichat calls the contractility of texture, and that this power is not limited to the period of life, but continues some time after death. It is equally ascertained that the capillaries are endowed during life with a peculiar degree of sensibility, which causes them to resist the intromission of such fluids as they are not destined to convey in the performance of their natural functions. This peculiar sensibility, by virtue of which the serous capillaries refuse, or contract against, the intromission of red blood, would seem to depend on the regular influx of the nervous influence. That this is the case appears highly probable, if not certain, from the different results arising from the forcible injection of fluids into the arteries in living and dead animals. "Push into the aorta of a living animal, by means of a syringe, different fine fluids, and you will never see them fill the capillary system, or issue by the exhalents;" when however the same experiment is performed on an animal soon after death, the fluid will be found to pass readily into the serous capillaries, and pass out by the exhalents, excretory ducts, &c. (*Bichat.*) Mr. Buniva's experiments, quoted by Bichat, with injections upon dead and living animals, illustrate this fact in a very striking manner. He fixed the pipe of a syringe into an artery of a living animal, and on endeavouring to force the fluid into the vessel he found very great resistance, the piston passing down very slowly, and only

Broussais and his followers are indeed fully sensible of the observation of Celsus: *Neque quicquam esse stultius quam quale quid vivo homine est, tale existimare esse moriente imo jam mortuo;* for where they fail in detecting a red and injected state of the mucous membrane of the bowels, they account for its absence by ascribing it to a *post mortem* change; thus availing themselves of this fact when it affords an argument in their favour; whilst they manifest an utter unwillingness to allow any importance to it when it is adduced against their doctrine.

It cannot indeed be presumed that the injected state of the mucous membrane of the intestinal tube, so often discovered in those who die from fevers, is always, or even generally, to be ascribed to a mere post mortem change; but that such changes do sometimes, nay often occur, and that they have been assumed as evidences of previous inflammation, there can exist but little doubt.

The first obvious effect of the remote febrific causes, consists almost universally in a diminution of the nervous energy, and consequently of the action of the heart and arteries; as is manifested by the weak and contracted pulse, the general langour and lassitude, the diminished temperature and the sense of chilwith the application of much force. On causing the animal to be suddenly killed by dividing the spinal marrow just below the occiput, the fluid passed rapidly out of the syringe into the artery, although but little force was applied. While the capillaries retained their full portion of vitality, they resisted the introduction of the fluid; but as soon as they had lost their sensibility in the death of the animal, they yielded like passive tubes to the fluid forced upon them by the *vis a tergo*. The application of these facts to the *post mortem* production of a red and injected state of the membraneous structures, especially the more vascular ones, is easily to be understood. So long as the serous capillaries retain their vitality, they resist the entrance into them of red blood. As soon however as their vital properties cease to exist, they lose the power of resisting the intromission of red blood —becoming in fact mere passive and yielding tubes. But as the arteries continue to contract on their contents some hours after the extinction of life, they must necessarily force the blood forward into the relaxed and unresisting capillary system, into which it will therefore be driven as into a sponge, and give to the more vascular structures the red and injected state so often found on post mortem examination, where no previous inflammation whatever existed.

liness which usher in all febrile affections. These initial phenomena of fever are especially conspicuous in intermittents, remittents, and in catarrhal affections. There is nothing in the character of these symptoms which can justify the inference that they are dependent on inflammation. "Inflammation," says Dr. Armstrong, "cannot exist in the cold stage of fevers, all the phenomena of which are directly opposed to inflammation." The course and phenomena of intermitting fevers present us indeed with insurmountable objections to the "*physiological doctrine.*" The periodicity of these fevers is strongly opposed to the idea of their immediate dependence on gastro-enteritis. It is indeed true that some affections of an inflammatory character have been known to recur in a strictly periodical manner; but such cases must be viewed as anomalies, and altogether contrary to the almost universal course and character of phlegmasial diseases. An inflammation which observes a perfect periodicity in its attacks, must be *sui generis.* If intermitting fever depend on inflammation of the mucous membrane of the alimentary canal, then must this inflammation be periodical, and therefore essentially distinct from the inflammation which produces *remitting* fever; for in this malady it must be continuous. These two forms of fever are however produced by the same remote cause; and we are therefore forced to admit, by the assumption of this doctrine, that the same remote cause is capable of producing two kinds of inflammation essentially distinct from each other. The character of the remedies too which have been found most effectual in arresting intermitting fever, is directly opposed to the idea that *gastro-enteritis* constitutes its proximate cause. Who can believe that quinine, arsenic, black pepper, and other remedies of a similar character are peculiarly calculated to cure inflammation of the mucous membrane of the alimentary canal? Indeed these very articles appear to be particularly dreaded by the disciples of this doctrine, on account of their tendency to create gastro-enteric irritation, and yet all experience goes to prove that they are decidedly the most prompt and valuable means for the cure of intermitting fever.

M. Broussais' theory of the mode in which the remote causes of febrile affections produce gastro-enteritis is gratuitous, and but little calculated to satisfy the understanding. "Every irritation," he says, "which is capable of producing a perception in the brain, passes back by the nerves *to be repeated in the mucous membranes.*" Thus if a person be innoculated with small-pox virus, the irritation of the primary pustule, or of the innoculated point, is conveyed to the brain, whence it is reflected by the nerves upon the mucous membranes of the alimentary canal, in which it establishes an inflammation. This intestinal inflammation constitutes the essential cause of eruptive fever, and the eruption itself is only a metatastic disorder of the cutaneous system. The assumption then, "that every irritation which is capable of producing a perception in the brain, is reflected by this organ to be *repeated in the mucous membranes* of the alimentary canal, forms the main principle in the Broussaian doctrine of the etiology of fever. That the mucous membranes of the intestinal tube possess a very wide sphere of sympathetic relations, is a fact indeed as undeniable as it is important in a pathological point of view. But that this structure constitutes a subordinate sensorium commune, to which all morbific impressions are especially conveyed, after having been perceived by the brain, is a position which all the zeal and ingenuity of its advocates have as yet failed, and I apprehend will ever fail to place upon that firm basis which it ought to have for a foundation of our pathological faith.

I do not mean to object to the general fact, that all impressions capable of ultimately exciting fever are in the first place communicated to the sensorium commune, and thence reflected throughout the system, and sometimes upon some particular organ or structure; but this reflected impression does not, it may be justly maintained, necessarily establish a focus of irritation, or always, nor even generally, fall especially upon the intestinal mucous membrane.

If the impressions of morbific causes are always transferred to the mucous membrane of the alimentary canal, the impressions of all agents, remedial as well as others, must of course be referred to the same structure. This, however, does not accord with the results of observations. When mercury is rubbed on the skin, the salivary glands, the gums, and the mucous membrane of the mouth receive the chief impressions excited by this agent. Will it be contended that a gastro-enteritis must be established before salivation can be produced? If opium be applied to any part of the body, the impressions are concentrated in the nervous centre. When cantharides are applied to the surface, the irritation is conveyed to the neck of the bladder, and not to the mucous membrane of the bowels, and yet fever will be the result. From these and many other similar facts that might be adduced, it is manifest that the supposed law of the animal economy, by which, as is alleged, all febrific impressions are reflected from the brain and *repeated* in the mucous membranes of the bowels, is gratuitous, or to say the least, highly improbable.

The fallacy of those doctrines which confine the primary inflammation to some one structure exclusively, is strikingly illustrated by the circumstance that different writers have fixed on *different* structures, as the parts primarily affected in fevers. Thus Clutterbuck maintains, with Broussais, that fever is always a purely symptomatic affection depending on a local inflammation pre-established by the febrific cause. He asserts that this primary inflammation is invariably located in the brain and its membranes, and adduces the phenomena discovered on post mortem examination, in testimony of the correctness of his doctrine. Broussais, on the other hand, asserts that the primary inflammation is not in the brain, but in the mucous membrane of the bowels, and appeals with equal confidence to the appearances exhibited on dissection for confirmation of *his* doctrine.

This discrepancy is in itself sufficient to show the weak foundation on which these two doctrines rest; for if the evidence afforded by autopsic inspection in relation to this subject were not extremely ambiguous, it would one may suppose, be impossible to draw from it conclusions so very discrepant, and yet so nearly equal in point of plausibility.

The advocates of the gastro-enteritic pathology of fever, place no inconsiderable reliance for support to their doctrine on, what they are pleased to assert, their superior success in the remediate management of fevers. Leeches, and an almost total abstinence from food, with cooling, acidulated, mucilaginous drinks, constitute nearly the whole of their remediate applications; and they claim for this mode of treatment, a greater success than that which they allow to others who pursue a more active course of treatment in fevers. It does not appear however that the golden age of medical success, so confidently promised by Broussais, on the introduction of his doctrine has as yet arrived;* nor do the statements which have been published in France, in reference to the comparative mortality under the Broussaian, and the other modes of treatment, give any confirmation to the claims of superior success set up for the former. We might however admit the excellence of the Broussaian mode of treating fevers, without yielding our assent to the correctness of the doctrine which alleges that the gastro-enteritis is primary where it *does* exist. There exists but little doubt in my mind, that in continued and remitting fevers, active purgation is not unfrequently carried to an injurious extent; for although we may, and, as I conceive, ought to reject the opinion that such fevers *depend* essentially on gastro-enteritis, yet it is beyond all doubt, that a very considerable degree of irritation, amounting in many instances to inflammation does often *supervene* during the progress of the disease as an *epiphenomenon*, and unconnected with the origination of the fever. In cases where such a condition of the

*In 1821, Broussais asserted, "that the tables of mortality declare in favour of the *new doctrine*, and that *its influence upon population would be more favourable than that of the introduction of vaccination.*" Unfortunately, however, this happy influence of the *nouvelle doctrine*, remains yet to be realized;* and the advocates of the doctrine may console themselves for the tardiness of this influence, with the certain prospect of not being very soon deprived of the opportunity of publishing their ordinary quantum of *post mortem* examinations.

*Refutation de la Doctrine de M. L. Doct. Broussais, Par L. Castel.

mucous membrane of the intestinal canal occurs during the course of the disease, the soothing treatment recommended by Broussais, would no doubt be much more salutary than the vigorous purgative plan so commonly pursued in this country and in England. Unquestionably intestinal irritation and inflammation perform an important part in febrile diseases. These conditions often arise as consequences of the general febrile action; and frequently, no doubt, also in consequence of harsh and repeated purgation, and the use of other irritating remediate agents. But it is equally probable that *gastro-enteritis* is often the result of the remora in the intestinal tube, of acrid and vitiated secretions, and other offensive materials, in consequence of withholding suitable laxatives, in the commencement and during the progress of the malady. Broussais has done much good, by awakening the attention of the profession to these pathological conditions; and thus added another proof of the fact, that new doctrines, though fundamentally erroneous, seldom fail to do some good, by directing the views of physicians to important circumstances which were previously overlooked or too much neglected.

In leaving this subject, I deem it right to observe, that however widely we may differ from Broussais in relation to the pathology of fever, all must admit that he has just and high claims to the respect and gratitude of the profession for the light which he has thrown on the nature, symptoms, and treatment of mucous intestinal inflammation, as well as on the physiological and morbid sympathies of the animal system.

Broussais is unquestionably one of the most enlightened and ingenious pathologists of the present day. His is now the only general doctrine which especially occupies the attention of the profession. Like all the preceding great doctrines in medicine, it is destined, perhaps, to culminate for a while in the firmament of our science, and to attract its host of worshippers; but, assuredly, sooner or later it must sink again, and add another to the long list of once highly favoured, but now exploded and neglected doctrines. That the Broussaian system contains much that is valuable, it would be unjust to deny; but to these concessions in its favour, there are, unless the majority of competent judges greatly err, offsets of no small moment. In relation to this doctrine, as indeed of every one else, it behoves us to embrace the useful and reject the false; in short, to adopt the good advice of Lucretius,

> "————doctrinam acri
> Judicio perpende; et si tibi vera videtur
> Dede manus—aut si falsa est, accingere contra."

